# Sporothriolide derivatives as chemotaxonomic markers for *Hypoxylon monticulosum*

**DOI:** 10.1080/21501203.2014.929600

**Published:** 2014-06-26

**Authors:** Frank Surup, Eric Kuhnert, Erik Lehmann, Simone Heitkämper, Kevin D. Hyde, Jacques Fournier, Marc Stadler

**Affiliations:** ^a^ Department Microbial Drugs, Helmholtz-Centre for Infection Research, Inhoffenstrasse 7, 38124Braunschweig, Germany; ^b^ German Centre for Infection Research (DZIF), Inhoffenstraße 7, 38124Braunschweig, Germany; ^c^ Institute of Excellence in Fungal Research and School of Science, Mae Fah Luang University, Chiang Rai57100, Thailand; ^d^ Las Muros, F-09420Rimont, France

**Keywords:** antibiotics, secondary metabolites, screening, chemotaxonomy, *Hypoxylon*, furofurandiones

## Abstract

During the course of a screening for novel anti-infective agents from cultures of tropical Xylariaceae originating from French Guiana and Thailand, pronounced antifungal activity was noted in extracts of cultures of *Hypoxylon monticulosum*. A bioassay-guided fractionation led to the known metabolite sporothriolide as active principle. In addition, three new derivatives of sporothriolide were isolated, for which we propose the trivial names sporothric acid, isosporothric acid and dihydroisosporothric acid. Their chemical structures were elucidated by high-resolution electrospray mass spectrometry in conjunction with two-dimensional nuclear magnetic resonance (2D-NMR) spectroscopy. From earlier studies on the biogenesis of the chemically similar canadensolides, we postulate that the new compounds were shunt products, rather than biogenetic precursors of sporothriolide. Interestingly, this compound class, as well as strong antifungal activities, was only observed in multiple cultures of *H. monticulosum*, but not in several hundreds of *Hypoxylon* cultures studied previously or concurrently. Therefore, sporothriolide production may constitute a species-specific feature with respect to *Hypoxylon* and the *Xylariaceae*, although the compound was previously reported from non-related fungal taxa.

## Introduction

Due to the ongoing development of resistances of human pathogens against common antibiotics, new drugs are urgently needed (Cooper and Shlaes 2011). Besides various chemical approaches, the exploration of natural sources for discovery of new anti-infective lead structures still seems most promising, because natural products have historically been the most prolific source for anti-infective drugs (Newman and Cragg 2012). Prominent examples are the beta-lactam antibiotics and many other useful compounds, which were mostly found during the course of empirical screening programs using fungal soil isolates.

**Figure 1.**
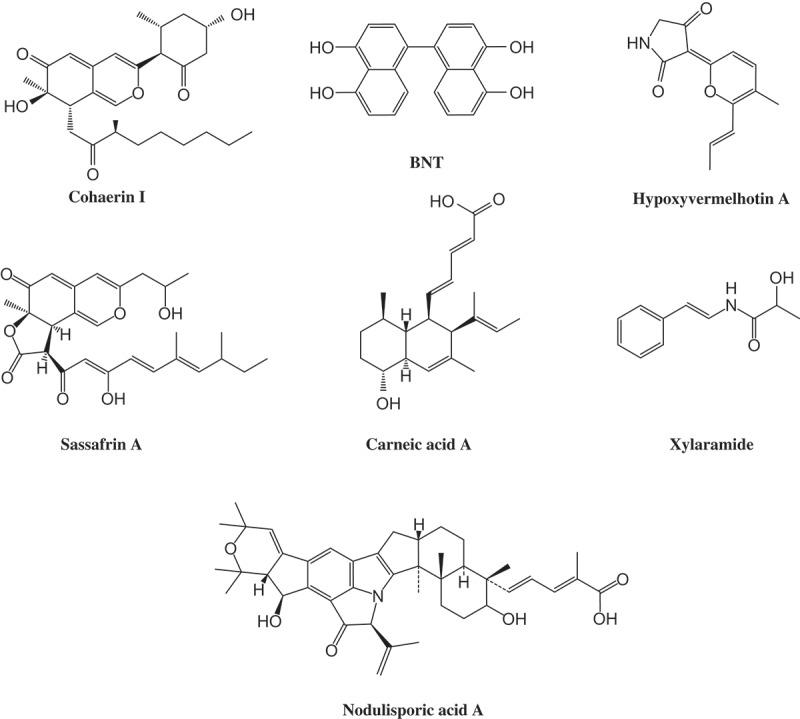
Structures of prominent secondary metabolites from Xylariaceae.

Through the availability of molecular phylogenetic and genomic data in conjunction with high-performance liquid chromatography (HPLC) profiling studies, ‘hotspots’ of metabolic diversity now can be identified within the fungal kingdom that can be circumscribed by taxonomic terms. One of the most prolific fungal families of secondary metabolite producers are the Xylariaceae, which are known for their high morphological and chemical diversity. Whalley and Edwards (1995 and references therein) already provided evidence for the high chemical diversity of secondary metabolites in this fungal family and pointed out their chemotaxonomic significance. Up to date, ca. 500 different secondary metabolites have been discovered, most of them exhibit some kind of biological activity. Interestingly, expression of secondary metabolism of these fungi may change completely as their life cycle’s progress (Stadler et al. 2006). The stromata, which grow mostly on dead wood, contain various cytotoxic agents, e.g., cohaerin azaphilones or tetramic acid hypoxyvermelhotin A (Figure 1; Surup et al. 2013, Kuhnert, Heitkämper et al. 2014) whereas the cultured mycelia produce various other metabolites, such as the potent antifungal agents hypoxysordarin and xylaral, or the antiparasitics PF-1022A and nodulisporic acid (Figure 1; Stadler and Hellwig 2005, Bills et al. 2012). Besides their bioactivity, secondary metabolites of Xylariaceae were proven to be a valuable tool for taxonomic purpose (Stadler and Hellwig 2005; Stadler and Fournier 2006; Stadler 2011). Some of these compounds are so unique in nature, that they are only produced by a certain species. Prominent examples are sassafrins in *Creosphaeria sassafras*, carneic acids in *Hypoxylon carneum* or hypoxyvermelhotins in *H. lechatii* (Figure 1; Quang et al. 2005, Quang et al. 2006, Kuhnert, Heitkämper et al. 2014). By means of high-performance liquid chromatography coupled with a diode array detector and a mass spectrometer (HPLC-DAD/MS) extracts from small amounts of stromata are sufficient to identify known compounds (Bitzer et al. 2007). The thereby generated chemoprofiles are specific for certain taxonomic levels down to species rank. However, some species within the Xylariaceae are devoid of secondary metabolites in fruiting bodies or produce only the ubiquitous BNT, e.g., all species of the genera *Camillea* and *Biscogniauxia* or some *Hypoxylon* species like *Hypoxylon monticulosum*. In these cases, chemotaxonomy is restricted to HPLC profiles derived from culture extracts. Despite the unlimited availability of strains and the very good possibility of upscaling, the evaluation of secondary metabolites from the Xylariaceae has been restricted so far to small molecules or those with conspicuous bioactivity. Bitzer et al. (2008) already showed that secondary metabolites derived from cultures are a valuable tool to resolve infrageneric and intergeneric relationships among various species within the Xylariaceae. However, the variety of taxonomic relevant marker metabolites is limited. The most striking one so far, nodulisporic acid produced by *Hypoxylon pulicicidum*, led to the development of a product candidate for an insecticidal drug used to treat animals (Bills et al. 2012). They also showed that nodulisporic acid appears restricted to *H. pulicicidum* and therefore serves as a taxonomic marker at the species level.

Due to the fact that the diversity of species within the Xylariaceae is the highest in tropical regions, the probability of finding producers of new bioactive secondary metabolites in these areas is considerably higher than in temperate regions. We therefore screened for the bioactivity of extracts prepared from around 50 submerged cultures derived from stromata of various Xylariaceae species originating from French Guiana. In this context, we found strong antifungal activity in extracts of *H. monticulosum*. The isolation and structural elucidation of the bioactive compound are reported here, and the chemotaxonomic relevance of these findings is discussed.

## Material & methods

### General

**Figure 2.**
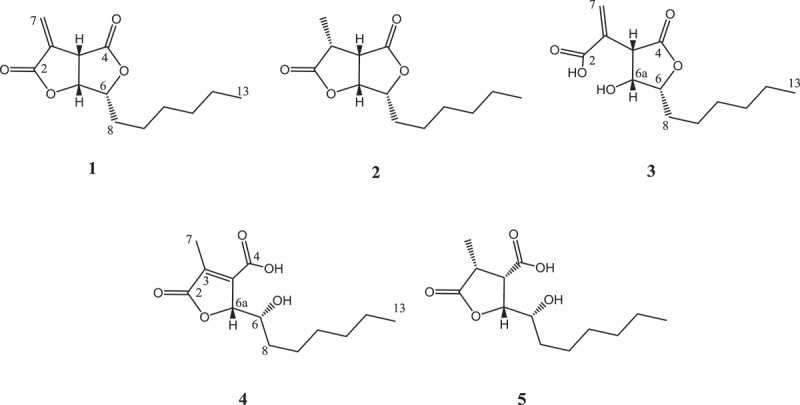
Structures of sporothriolide (**1**), dihydrosporothriolide (**2**), sporothric acid (**3**), isosporothric acid (**4**) and dihydroisosporothric acid (**5**) isolated from submerged cultures of *Hypoxylon monticulosum* (MUCL 54604).

If not indicated otherwise, solvents were obtained in analytical grade from J.T.Baker (Deventer, Netherlands) or Merck (Darmstadt, Germany). Bold numbering refers to the chemical structures depicted in Figure 2. All scientific names of fungi follow the entries in Mycobank (www.mycobank.org). Reference specimens are housed in LIP (University of Lille, France) or Mae Fah Luang University (MFLU) herbarium and corresponding reference cultures have been deposited with MUCL (Louvain, Belgium), MFLU or DSMZ (Braunschweig, Germany). Acronyms of herbaria and culture collections are given as recommended in Index Herbariorum (http://sciweb.nybg.org/science2/IndexHerbariorum.asp).

### Fungal material

**Figure 3.**
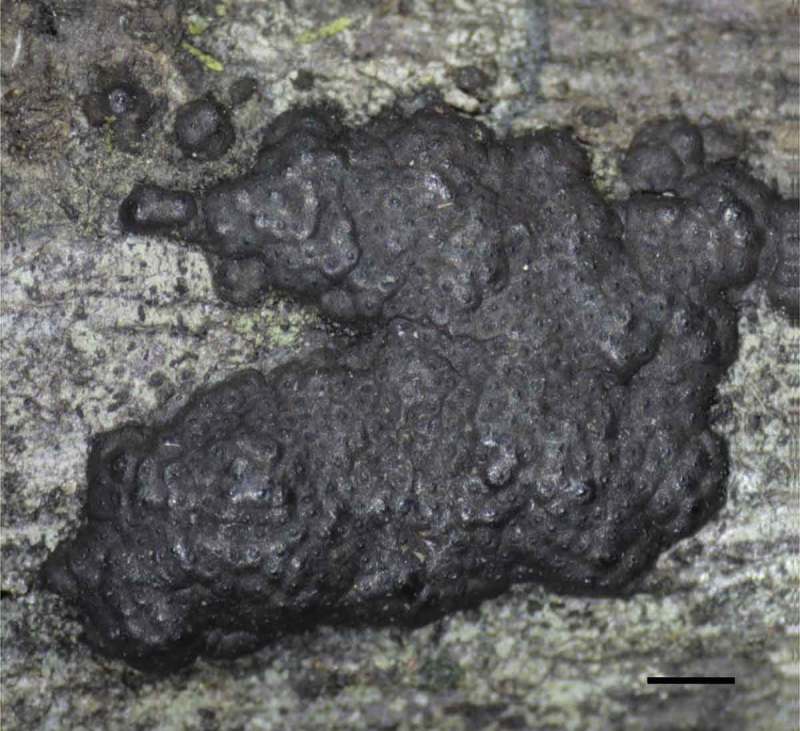
Stroma of *Hypoxylon monticulosum* GYJF12243 on natural substrate. Scale is indicated by bar: 1 mm.

The screening was carried out on cultures derived from 27 strains of various Xylariaceae species (LIP), including 12 *Hypoxylon* species, 7 *Annulohypoxylon* species, 2 *Phylacia* species and 1 *Biscogniauxia* species, which were collected in 2012 in French Guyana. Among these the *H. monticulosum* strain GYJF12243 (Figure 3) and two additional specimens of the same species collected in 2013 in Thailand (MFLU 13–0356, MFLU 13–0358) were chosen for further evaluation. Cultures (MUCL 54604, MFLUCC 13–0593, MFLUCC 13–0595) were derived from multispore isolation of the respective fruiting bodies as described in Kuhnert, Fournier et al. (2014). Strains were determined by morphological characters and supported by molecular data of the ITS region (Genbank Acc. No.: KJ810554, KJ810555, KJ810556) according to Kuhnert, Fournier et al. (2014). To clarify the degree of relationship between *H. monticulosum* and the original producer strain of sporothriolide, ‘*Sporothrix* sp.’ (DSM 28461, Krohn et al. 1994), the ITS region of the latter was also sequenced.

### Cultivation and extraction

All strains were grown in submerged cultures (500-ml Erlenmeyer flasks) containing 200 ml of YMG media (10 g l^−1^ malt, 4 g l^−1^ glucose, 4 g l^−1^ yeast extract, pH 6.3). For scale-up, a batch of 19 shake flasks of strain MUCL 54604 was inoculated. Furthermore, the latter strain was also cultivated in 20 flasks, each containing 200 ml of ZM½ media (5 g l^−1^ molasses, 5 g l^−1^ oat meal, 1.5 g l^−1^ glucose, 4 g l^−1^ sucrose, 4 g l^−1^ mannitol, 0.5 g l^−1^ edamine, 0.5 g l^−1^(NH_4_)_2_SO_4_, 1.5 g l^−1^ CaCO_3_, pH 7.2). Fungal cultures were incubated at 23°C on a rotary shaker at 140 min^−1^. Fermentations were aborted after free glucose was consumed (Stadler et al. 2001).

The culture broth of the large scale production was (20 × 200 ml) combined and afterwards biomass was separated from the media by vacuum filtration. The mycelia were extracted in both scales with 200 and 1000 ml acetone, respectively. The organic extract was then filtered and evaporated. The remaining water phase was extracted with the same amount of ethyl acetate in a separating funnel. The organic phase was then mixed with sodium sulfate, filtered and again evaporated to yield dry mycelia extracts (ME). In small scale, the supernatant was mixed with 200 ml ethyl acetate for 15 min on a magnetic stirrer and additionally extracted in an ultrasonic bath for 15 min at 40°C. The organic phase was separated and proceeded as for the ME. In the case of the 4-l fermentation, the supernatant was mixed with 80 g adsorbent resin (Amberlite XAD™-16N) and incubated overnight. The Amberlite was then filtered and eluted with 800 ml methanol. The extract was also evaporated until a water phase remained. The aqueous phase was extracted with ethyl acetate and processed as described for the ME. The extracts of the 29 strains were tested for activity against *Bacillus subtilis* (DSM 10), *Escherischia coli* (DSM 1116), *Pichia anomala* (DSM 6766) and *Mucor plumbeus* (MUCL 49355) as described by Halecker et al. (2014).

### Isolation of the bioactive compounds

The extract derived from the supernatant of the strain MUCL 54604 grown in YMG media showed the strongest antifungal activity and was therefore first processed. The extract was filtered through a RP solid phase cartridge (Strata-X 33 µm, Polymeric Reversed Phase; Phenomenex, Aschaffenburg, Germany) to yield 152 mg crude extract. The bioactive compound was identified by means of bioassay-guided fractionation and later isolated by preparative reversed phase liquid chromatography (PLC 2020, Gilson, Middleton, USA) under the following conditions: A VP Nucleodur 100–5 C18 ec column (250 × 21 mm, 5 µm; Macherey-Nagel) was used as stationary phase. The mobile phase was composed of deionised water (Milli-Q, Millipore, Schwalbach, Germany) with 0.05% TFA (solvent A; Roth) and acetonitrile with 0.1% acetic acid (solvent B). A flow rate of 15 ml min^−1^ was used for the following gradient: 40–80% solvent B in 30 min, afterwards linear gradient to 100% solvent B in 10 min, thereafter isocratic conditions at 100% for 10 min. UV detection was carried out at 210 nm and 254 nm and fractions were collected and combined according to the observed peaks. In addition to the active compound **1** (54.7 mg) at a retention time (Rt) of 23–25 min, compound **4** was yielded in pure amounts (2.7 mg) at Rt = 15 min. The ME derived from both media was fractionized as described for the supernatant extract. Compound **5** was isolated in pure state (0.5 mg) at Rt = 12 min from ME of the YMG fermentation. Compound **3** (1.0 mg, Rt = 11 min) and compound **2** (4.2 mg, Rt = 23 min) were yielded from ME of the ZM½ fermentation.

*Sporothriolide* (**1**): colorless oil; [α]^25^_D_ −144 (*c* 3.3, CHCl_3_); ^1^H NMR, ^13^C NMR see Tables 1 and 2; high-resolution electrospray mass spectrometry (HR-ESI-MS) *m/z* 239.1274 [M + H]^ +^ (calcd for C_13_H_19_O_4_, 239.1278); spectral data are in good agreement with Krohn et al. (1994).

**Table 1.** ^1^H NMR data for metabolites **1**–**5** (CDCl_3_, 700 MHz).

#^a^	1	2	3	4	5
3		3.05, dq (10.1, 7.5)			2.99, dq (11.0, 7.1)
3a	4.00, dt (6.7, 2.1)	3.44, dd (10.1, 6.0)	3.82, dd (3.9, 1.4)		3.23, dd (11.0, 9.0)
6	4.64, ddd (8.0, 6.3, 4.6)	4.50, ddd (8.0, 6.2, 3.9)	4.51, ddd (8.6, 5.2, 1.4)	4.25, dt (7.0, 1.5)	3.71, m
6a	5.14, dd (6.7, 4.6)	5.01, dd (6.0, 3.9)	4.30, dd (3.9, 1.3)	5.06, qd (2.1, 1.5)	4.47, dd (9.0, 2.2)
7	6.46, d (2.1) 6.15, d (2.1)	1.47, d (7.5)	6.78, s 5.95, s	2.24, d (2.1)	
8	1.86, m	1.92, m; 1.81, m	1.80, m; 1.69, m	1.73, m	1.82, m
9	1.50, m	1.50, m	1.54, m; 1.38, m	1.50, m	1.46, m
10	1.37, m	1.37, m	1.32, m	1.34, m	1.29, m
11	1.30, m	1.30, m	1.27, m	1.28, m	1.25, m
12	1.31, m	1.31, m	1.28, m	1.29, m	1.26, m
13	0.88, t (7.0)	0.88, t (7.0)	0.88, t (7.0)	0.88, t (7.0)	0.88, t (7.0)

Note: ^a^For clarity the numbering of **3**–**5** was based on compound **1**.

**Table 2.** ^13^C NMR data for metabolites **1**–**5** (CDCl_3_, 175 MHz).

#^a^	1	2	3	4	5
2	167.5	176.2	163.5	173.1	176.7
3	129.9	36.8	128.5	140.2	39.2
3a	46.2	44.7	50.0	144.7	48.2
4	172.1	172.1	171.3	164.8	171.9
6	82.8	81.7	79.1	70.1	71.0
6a	77.2	78.1	66.5	83.1	80.6
7	127.4	11.1	134.6	11.2	14.8
8	28.9	28.9	31.0	34.4	34.0
9	25.4	25.3	25.1	25.8	25.7
10	29.0	29.0	29.0	29.1	29.0
11	31.6	31.6	31.6	31.7	31.8
12	22.5	22.6	22.6	22.6	22.6
13	14.1	14.2	14.1	14.1	14.2

Note: ^a^For clarity the numbering of **3**–**5** was based on compound **1**.

*Dihydrosporothriolide* (**2**): colorless oil; [α]^25^_D_ +116 (*c* 0.1, CHCl_3_); ^1^H NMR, ^13^C NMR see Tables 1 and 2; HR-ESI-MS *m/z* 241.1429 [M + H]^ +^ (calcd for C_13_H_21_O_4_, 241.1434); spectral data are in good agreement with Krohn et al. (1994).

*Sporothric acid* (**3**): colorless oil; [α]^25^_D_ +14 (*c* 0.1, CHCl_3_); ^1^H NMR, ^13^C NMR see Tables 1 and 2; IR (KBr) 2957, 2928, 2858, 1762, 1738, 1627, 1384, 1272, 1189 cm^−1^; HR-ESI-MS *m/z* 257.1382 [M + H]^ +^ (calcd for C_13_H_21_O_5_, 257.1384).

*Isosporothric acid* (**4**): colorless oil; [α]^25^_D_ −18 (*c* 0.5, CHCl_3_); ^1^H NMR, ^13^C NMR see Tables 1 and 2; IR (KBr) 2957, 2927, 2857, 1749, 1728, 1213, 1114, 719 cm^−1^; HR-ESI-MS *m/z* 257.1384 [M + H]^ +^ (calcd for C_13_H_21_O_5_, 257.1384).

*Dihydroisosporothric acid* (**5**): colorless oil; [α]^25^_D_ +16 (*c* 0.04, CHCl_3_); ^1^H NMR, ^13^C NMR see Tables 1 and 2; IR (KBr) 2956, 2929, 2858, 1723, 1384, 1294, 1185, 1027 cm^−1^; HR-ESI-MS *m/z* 259.1544 [M + H]^ +^ (calcd for C_13_H_23_O_5_, 259.1540), 281.1363 [M + Na]^ +^ (calcd for C_13_H_22_O_5_Na, 281.1359).

### Hydrogenation of sporothriolide (1) to dihydrosporothriolide (2)

A solution of 12.5 mg of sporothriolide (**1**) in 2 ml of methanol was stirred in the presence of 5 mg of 10% Pd/charcoal under an atmosphere of hydrogen (1 atm) at ambient temperature for 2 h. The solution was centrifuged (14600 rpm, 5 min) and purified by preparative HPLC to yield 6.9 mg (55%) of **2**.

### Structure elucidation

Optical rotations were determined with a PerkinElmer 241 polarimeter, UV–Vis spectra were recorded with a Shimadzu (Kyoto, Japan) UV–Vis spectrophotometer UV-2450, IR spectra were measured with Spectrum 100 FTIR spectrometer (Perkin Elmer). NMR spectra were recorded with a Bruker (Bremen, Germany) Ascend 700 spectrometer with a 5 mm TXI cryoprobe (^1^H 700 MHz, ^13^C 175 MHz). Electrospray ionization mass spectrometry spectra were obtained with an ion trap MS (Amazon, Bruker), high resolution electrospray ionization mass spectrometry spectra were obtained with a time-of-flight MS (Maxis, Bruker) as described by Pažoutová et al. (2013). For HPLC-based dereplication, data from the cited previous studies on Xylariaceae, recorded by the method described in general by Bitzer et al. (2007), including various standards of previously published metabolites, were used in conjunction with the Dictionary of Natural Products (Dictionary… 2013).

### Bioactivity assay

Minimum inhibitory concentrations (MIC) were recorded in a serial dilution assay with pure substances using various test organisms for antibacterial and antifungal activity (Halecker et al. 2014). Cytotoxicity was assayed in a similar manner as described by Herrmann et al. (2012) employing the cell lines HCT-116 (human colon carcinoma), CHO-K1 (Chinese hamster ovary) and U-2 OS (human bone osteosarcoma).

## Results

### Structure elucidation

The bioactive metabolite **1** was isolated from the crude extract by preparative HPLC. The molecular formula of C_13_H_18_O_4_ was deduced from an [M + H]^ +^ molecular ion at *m/z* 239.1274 in the HR-ESI-MS spectrum. The proton and heteronuclear single quantum coherence NMR spectra of **1** revealed the presence of an exocyclic methylene (*δ* 6.46, 6.15), three coupled methines (*δ* 5.14, 4.64, 4.00), five methylenes (*δ* 1.30–1.86) and a methyl group (*δ* 0.88). A literature search within the Chapman and Hall Dictionary of Natural Products (Dictionary… 2013) and Chemical Abstracts Plus (SciFinder Scholar) databases unambiguously confirmed that the compound **1** is identical to sporothriolide (**1**) (Krohn et al. 1994).

As by-products of the HPLC fractionation multiple derivatives of **1** were obtained. The first is a metabolite with a molecular formula of C_13_H_20_O_4_. The proton NMR of **2** was very similar to the one of **1** except the replacement of the exomethylene by a methyl group and the appearance of an additional methine signal. The data are consistent with the formal hydrogenation of **1** to its 3,7-dihydro analogue. As demonstrated by the observed chemical shifts and coupling constants for H-3 (*δ* 3.05, dq, *J*_3,3a_ = 10.1 Hz, *J*_3,7_ = 7.5 Hz) and H-3a (*δ* 3.44, dd, *J*_3,3a_ = 10.1 Hz, *J*_3a,6a_ = 6.0 Hz), the stereo configuration of **2** was assigned as (3*R*,3a*S*,6*R*,6a*R*), which is the same as those reported for the synthetic dihydrosporothriolide (Krohn et al. 1994), differing from the previously isolated dihydrosporothriolide from *Xylaria* (Isaka et al. 2010) in having a (3*S*,3a*S*,6*R*,6a*R*) stereochemistry. Rotating frame nuclear overhauser effect spectroscopy (ROESY) correlations from H-3 to H-3a and H-6a furthermore supported the *cisoidal* configuration of H-3, H-3a and H-6a. To obtain more material of this minor metabolite, main metabolite sporothriolide (**1**) was hydrogenated according to Krohn et al. (1994).

Metabolite **3** showed a [M + Na]^ +^ molecular ion at 257.1384, which indicated a molecular formula of C_13_H_20_O_5_. This was a formal addition of water compared to the major metabolite **1**. The main spectral difference to sporothriolide (**1**) was the upfield shift of H-6a in the ^1^H NMR spectrum. This was consistent with an opening of the lactone ring bearing the exocyclic methylene moiety. A ROESY correlation between H-3a and H-6a indicates an *cisoidal* configuration between the two protons, therefore the structure was established as 2-[(3*S*,4*R*,5*R*)-5-hexyl-4-hydroxy-2-oxotetrahydrofuran-3-yl]prop-2-enoic acid. Due to its acidic nature the name sporothric acid was suggested for **3**.

For metabolite **4** a molecular formula of C_13_H_20_O_5_ was concluded from its [M + Na]^ +^ adduct at 257.1384. The proton NMR indicated a ring opening due to the high field shift of H-6 compared to sporothriolide (**1**). Furthermore, carbon and heteronuclear multiple-bond correlation NMR spectra demonstrated that C-3 and C-3a were quaternary sp^2^ atoms. Due to a common biosynthesis of **1**–**5** a 6*R* configuration can be assumed for **4** and **5**, so the structure of metabolite **4** was determined to be (2*R*)-2-[(1*R*)-1-hydroxyheptyl]-4-methyl-5-oxo-2,5-dihydrofuran-3-carboxylic acid and named isosporothric acid.

Compound **5** possesses the molecular formula C_13_H_22_O_5_, which was deduced from its [M + Na]^ +^ adduct at 281.1363, is implying a formal addition of H_2_O compared to **2**. The key difference of spectroscopic data, when compared to those of **2**, was the upfield shift of H-6 (δ 3.71) in the ^1^H NMR spectrum. Therefore a ring opening of lactone ring could be concluded. ROESY correlations of H-3 with both H-3a and H-6a established the structure of **5** as (2*R*,3*S*,4*R*)-2-[(1*R*)-1-hydroxyheptyl]-4-methyl-5-oxotetrahydrofuran-3-carboxylic acid. The name dihydroisosporothric acid was proposed for **5**.

### Evaluation of sporothriolide as a marker metabolite for H. monticulosum

The *H. monticulosum* strains from Thailand (MFLUCC 13–0593, MFLUCC 13–0595) were evaluated for the presence of sporothriolide. HPLC profiling of the crude extracts from submerged cultures revealed significant amounts of sporothriolide in both strains. Furthermore, the crude extracts showed bioactivity against *Mucor plumbeus*. The original producer strain ‘*Sporothrix* sp.’ was also reevaluated for its ability to produce the compound by applying the same fermentation parameters as for the *H. monticulosum* strains. Sporothriolide could not be unambiguously detected. Furthermore, the molecular data of the ITS region reveal that the species belong most likely to the genus *Emericellopsis* (Hypocreales; asexual state *Acremonium*) rather than to the more distantly related genus *Sporothrix* (Ophiostomatales). Therefore, it is not clear if this strain is still the original producer strain or a contamination. Due to this fact the fermentation was not repeated with the parameters described by Krohn et al. (1994).

### Bioactivity of sporothriolide

Sporothriolide (**1**) was found devoid of activity against Gram-positive and Gram-negative bacteria. Furthermore, no cytotoxic effects on the cell lines HCT-116, CHO-K1 and U-2 OS were observed. Strong antifungal activity was found on tested filamentous fungi and yeasts, including *Candida albicans* (Table 3). Metabolites **2** and **4** showed no activity, **3** and **5** were not tested due to the fact that only minor amounts were available. Our results of the bioactivity of sporothriolide (**1**) and dihydrosporothriolide (**2**) resemble the study of Krohn et al. (1994) which used an agar plate diffusion assay. The strong antifungal activity of furofurandiones has furthermore previously been described for canadensolide (**6**) (McCorkindale et al. 1968), ethiosolide (Atienza et al. 1992) and avenaciolide (Brookes et al. 1963).

**Table 3.** Minimal inhibitory concentrations (MIC) of sporothriolide (**1**), dihydrosporothriolide (**2**) and isosporothric acid (**4**), all solved in MeOH, and control drugs. [a] Oxytetracyclin hydrochloride, [b] Gentamycin, [c] Nystatin, [d] Amphotericin B, [e] Polymyxin B sulphate; -: no inhibition; n.t.: not tested. The cell density was adjusted to 8 × 10^6^ cells/ml. * spores from agar plate were applied without justification.

Test organisms	MIC [µg/ml]			
	1	2	4	Reference
Mucor hiemalis* (DSM 2656)	4.2	n.t.	n.t.	5.25 ^[c]^
*Candida albicans* (DSM 1665)	16.6	—	—	8.3–33.3 ^[c]^
*Nematospora coryli*	8.3	n.t.	n.t.	67.0 ^[c]^
*Pichia anomala* (DSM 8766)	33.3	—	—	8.3–33.3 ^[c]^
*Rhodotorula glutinis* (DSM 10134)	16.6	n.t.	n.t	16.7 ^[c]^
*Schizosaccharomyces pombe* (DSM 70572)	8.3	n.t.	n.t.	41.5 ^[c]^
*Trichosporon oleaginosus* (DSM 11815)	16.6	n.t.	n.t.	4.2 ^[c]^
*Bacillus subtilis* (DSM 10)	—	—	—	33.3 ^[a]^
*E. coli* (DSM 1116)	—	—	—	0.83 ^[a]^
*Pseudomonas aeruginosa* (DSM 50071)	—	—	—	16.6 ^[b]^

## Discussion

Furofurandiones are a common class of fungal *α*–methylene-*γ*-butyrolactones (Kitson et al. 2009). The metabolites can retro-biosynthetically be cleaved and they are probably being generated from a fatty acid and a C_3_ moiety, which originates from oxaloacetate (Chesters and O´Hagan 1997). Those natural products resulting from condensation of the *α*-position of the fatty acid are referred as type A metabolites and include sporothriolide (Krohn et al. 1994), canadensolide (McCorkindale et al. 1968) and xylobovide (Abate et al. 1997). In contrast, in isomeric type B metabolites like ethisolide (Atienza et al. 1992) and avenaciolide (Brookes et al. 1963) the C_3_ unit is linked to the *β*-position of the fatty acid chain.

**Figure 4.**
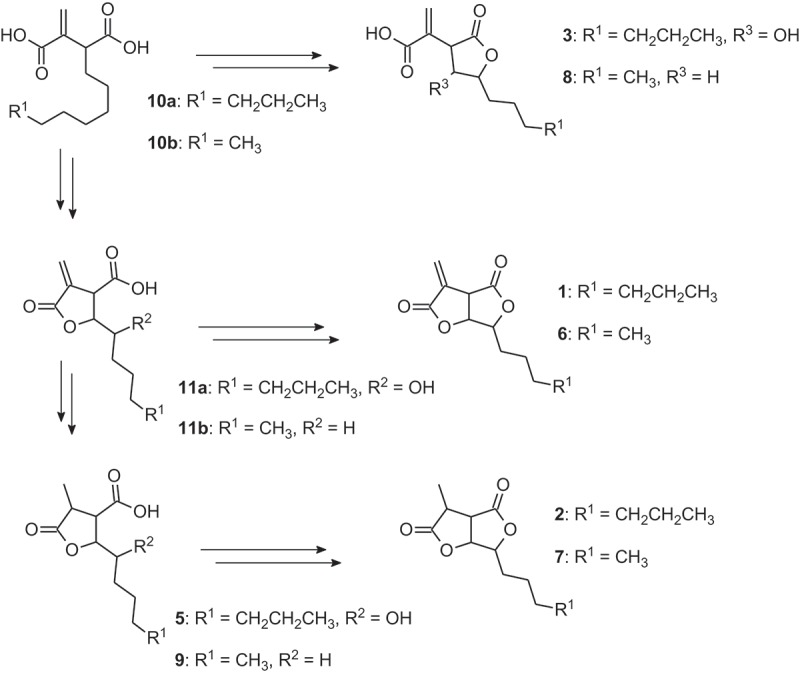
Hypothetical biosynthesis of sporothriolide (**1**), canadensolide (**6**) and their respective derivatives. Their relationship is indicated by feeding experiments with canadensolide derivatives by Hayes (1982).

Although metabolites **3**–**5** might be artifacts from saponification of **1**, their presence in the crude extract of *H. monticulosum* as well as previous reports on the chemically similar canadensolide derivatives indicate an origin as natural products. Apart from canadensolide and dihydrocandensoide, a number of related metabolites were isolated from cultures of *Penicillium canadense* (Hayes 1982, and references cited therein). The biosynthesis of canadensolide (**6**) and its derivatives dihydrocanadensolide (**7**), canadensic acid (**8**), isocanadensic acid and dihydroisocanadensic acid (**9**) was investigated with extensive labeling studies using radiolabeled or ^13^C-labeled precursors. These experiments indicated that **7**–**9** are not precursors of **6**, but are generated from common precursors **10b** and **11b**. According to the structural similarity of sporothriolide to candensolide, we can deduce the biosynthetic relationship between metabolites **1**–**5** as shown in Figure 4. Therefore **2**–**5** can be considered as shunt products, but not as precursors of sporothriolide (**1**).

Furofurandiones were previously described from two different strains of the genus *Xylaria*. Xylobovide was produced in cultures of *Xylaria obovata* (Abate et al. 1997), whereas a stereoisomer of dihydrosporothriolide was reported from cultures of two endophytes tentatively assigned to the genus *Xylaria* merely based on ITS sequencing (Isaka et al. 2010). However, this is the first report of these compounds being produced by *Hypoxylon* sp. So far the metabolite was only found in cultures of *H. monticulosum*. This observation becomes valuable due to the fact that stromatal extracts of the species contain only a minor unidentified compound, which is produced by young stromata. Older material is often devoid of this metabolite. Bitzer et al. (2008) already established strong correlations between chemotypes according to secondary metabolite production in culture and molecular phylogenetic data. They assigned *H. monticulosum* in the chemotype B, which is defined by the presence of 5-methymellein and the lack of other marker metabolites like mellein, 1–8-naphthol or isosclerones. However, the strain of *H. monticulosum* they used meanwhile turned out to be contaminated by an *Annulohypoxylon* species (M.S. & D. Persoh, unpublished data). Our new isolates of *H. monticulosum* were found devoid of 5-methylmellein.

From our experience, by far not all metabolites of Xylariaceae cultures can be used as chemotaxonomic markers, as the occurrence of certain compounds may be strongly dependent on the culture conditions, and some metabolites may even be strain specific. Therefore, we cannot exclude that the strains of species other than *H. monticulosum* we studied previously or concurrently do not contain traces of sporothriolide. However, sporothriolide was produced under various conditions in *H. monticulosium*, even when applying completely different media. Therefore, it might be a species-specific chemotaxonomic marker compound.

As the original producer strain was identified as *Sporothix* sp. a close relationship between this strain and *H. monticulosum* was expected due to the fact that sporothrix-like conidial states were defined as a subtype of nodulisporium-like asexual states of *Hypoxylon sensu* Ju and Rogers (1996). They also represent the asexual morphs of other genera in the hypoxyloid Xylariaceae, including *Daldinia* (Stadler et al. 2014), *Phylacia* (Bitzer et al. 2008), *Rhopalostroma* (Stadler, Fournier, Gardt, et al. 2010), *Ruwenzoria* (Stadler, Fournier, Læssøe, Decock, et al. 2010) and *Thamnomyces* (Stadler, Fournier, Læssøe, Chlebicki, et al. 2010). Several of these species are known to have asexual morphs reminiscent of *Sporothrix*. However, the molecular phylogenetic data generated from the original sporothriolide producer strain clearly revealed it to belong to the Hypocreales. Aside from the above cited papers on hypoxyloid genera, even the strains obtained by Bills et al. (2012), Fournier, Köpcke, et al. (2010), Fournier, Stadler et al. (2010), Fournier et al. (2011), Læssøe et al. (2013), Persoh et al. (2009) were all checked for antifungal effects and by chemotaxonomic methodology, but no sporothriolide was detected by HPLC–MS and prominent antifungal effects of the *Xylaria* sp. studied were always due to the overproduction of cytochalasins and other compounds.

Specific antifungal activity with lack of cytotoxicity on mammalian cell lines or antibacterial activity is a distinctive screening signal. Frequently, antifungal compounds affect the same targets in other eukaryotic cells (Roemer et al. 2011), and even in the case for a drug with a nonspecific mode-of-action like nystatin (Zheng and Audus 1994). Due to nystatin’s toxicity, it can only be applied as an oral or topical treatment. Forthcoming studies on the mode-of-action of sporothriolide might reveal a new target for antifungal treatment with reduced side effects.
